# Clinical Patterns and Treatment of Pediatric Facial Fractures: A 10-Year Retrospective Romanian Study

**DOI:** 10.3390/children10050800

**Published:** 2023-04-28

**Authors:** Raluca Iulia Juncar, Abel Emanuel Moca, Mihai Juncar, Rahela Tabita Moca, Paul Andrei Țenț

**Affiliations:** 1 Department of Dentistry, Faculty of Medicine and Pharmacy, University of Oradea, 10 Piața 1 Decembrie Street, 410073 Oradea, Romania; 2 Doctoral School of Biomedical Sciences, University of Oradea, 1 Universității Street, 410087 Oradea, Romania

**Keywords:** pediatric facial fractures, clinical patterns, treatment, Romania

## Abstract

Pediatric facial fractures have different clinical patterns and require different therapeutic approaches in comparison with those of facial fractures that occur among adults. The aim of this study was to describe the main clinical characteristics of pediatric facial fractures (such as fracture location, fracture pattern, treatment, complications and evolution) in a group of pediatric patients from NW Romania. This research was a retrospective study that was conducted for 10 years in a tertiary hospital for oral and maxillofacial surgery from NW Romania. A total of 142 pediatric patients were included in this study, with ages between 0 and 18 years. Mandibular (66.2%), midface (25.4%) and combined fractures (8.5%) were identified, and patients from the 13–18 years age group were more frequently affected by facial fractures (78.9%). Most of the diagnosed fractures among all three types of fractures were total fractures, and most mandibular (92.6%) and midface (80.6%) fractures were without displacement. Hematomas, lacerations and abrasions were identified as associated lesions. Patients with associated lesions were more frequently associated with combined fractures or midface fractures than mandibular fractures. The instituted treatment was, in general, orthopedic, for all three types of fractures (mandibular—86.2%; midface—91.7%; combined—66.7%). Most fractures, mandibular (96.8%), midface (100%) and combined (91.7%) fractures, had a favorable evolution. Most fractures did not present any complications at the follow-up. Pediatric facial fractures have unique patterns and must be treated with caution, considering the particularities of pediatric facial anatomy.

## 1. Introduction

Facial fractures are frequently encountered in emergency departments [1], and they are traumas that can cause disabilities [2]. For the proper treatment of facial fractures, interdisciplinary collaboration is needed, involving teams of plastic surgeons and maxillofacial surgeons [2]. Although they are less common among children, and more frequent among adults [3], pediatric facial fractures may have a negative impact on children’s development [4], are frequently associated with severe injuries and can cause morbidity and disability [5]. In order to reduce the negative effects and to achieve an optimal therapeutic result, it is necessary that the initial evaluation is thorough, the established diagnosis is correct and the treatment is instituted immediately [4].

Pediatric facial fractures have a relatively low incidence, ranging from 4.6% [6] to 14.7% [7] among patients under the age of 18, and have a complex etiology, which varies depending on the age of the child, but accidents occurring during the playing of different sports [8], traffic accidents [9] and fall injuries [10] are among the etiological factors that are most frequently incriminated. Despite the relatively low incidence, pediatric facial fractures have a significant morbidity [6] because due to the anatomical particularities of the pediatric patient [11], the force required to produce a pediatric facial fracture is much higher than the one required to produce a similar fracture in adult patients [7]. Pediatric facial fractures must be treated differently to those that occur among adult patients, both due to the different anatomy of the child, but also due to the impact on future growth and development [12]. In early childhood, the midface is protected by the mandible and the forehead, which can be more prominent. This characteristic makes midface fractures rarer until the age of six [5], but as the child grows, the midface becomes more prominent, and the incidence of midface fractures also increases [12]. Pediatric facial fractures generally occur without displacement due to them having flexible sutures and a more flexible facial skeleton. The strength of the mandible and maxilla is also increased by unerupted permanent teeth [13]. Pneumatization and the development of the paranasal sinuses ensure additional resistance to fractures among children. The ethmoid and maxillary sinuses begin to develop in utero, and at birth, the maxillary sinuses appear as small sacs in the mesenchyme of the lateral nasal wall [14]. The ethmoid sinuses almost reach their final size at the age of 12 years, and the maxillary sinuses mature at 9 years of age [15]. The sphenoid bone begins to pneumatize at the third year of life, and the development of the nose and paranasal sinuses continues until they reach their final size, around the age of 18 [14].

These anatomical particularities also influence diagnosis because pediatric facial fractures have distinct imaging characteristics [16]. Two-dimensional radiography offers limited information that can be used for the diagnosis of pediatric facial fractures, especially for midface and condylar fractures, but panoramic radiography is useful for the initial imaging evaluation when a fracture in the body of the mandible is suspected [16]. The best imaging modality for the diagnosis of facial fractures is computed tomography (CT), which allows the precise visualization of anatomical details necessary for the treatment of fractures [16], and in children, the specificity and sensitivity of head and face CT for detecting facial fractures is high: up to 100% [17]. However, small children are often uncooperative, and the imaging examination can only be performed under sedation [18]. In addition, CT requires a higher dose of radiation [16]. Due to these disadvantages, the diagnosis of pediatric facial fractures is often based solely on clinical examination [19].

The treatment of pediatric facial fractures varies based on the confirmed diagnosis and must account for children’s and adolescents’ active growth [20]. Generally, some principles of the reduction and stabilization of oral and maxillofacial fractures that apply to adults can also be applied to pediatric patients [20], but the different anatomical characteristics of children, as well as their active growth, must be considered when opting for a certain therapeutic approach [21]. In children, a conservative treatment is generally preferred, but when the fractures are severe, rigid fixation can be used for short periods of time in order not to compromise the developing dentition or skeletal growth [20]. The clinical characteristics of pediatric facial fractures differ among the various populations studied [22], and knowledge of these characteristics is essential in order to ensure a good prevention and an optimal therapeutic management. The authors were unable to find a publication that described the clinical features of pediatric facial fractures in the area that was researched up until the time this research was conducted.

The aim of this study was to describe the main clinical patterns of pediatric facial fractures (such as fracture location, fracture pattern, treatment, complications, evolution) among a sample of children and adolescents from NW Romania.

## 2. Materials and Methods

### 2.1. Ethical Considerations

The study was authorized by the University of Oradea’s Ethics Committee (IRB No. 3402/15.04.2018) and was carried out in accordance with the guidelines outlined in the 2008 Declaration of Helsinki and its following amendments. The legal guardians of the minors who participated in the study signed consent forms allowing the anonymous use of their medical information. Patients aged 18 had the possibility to complete a consent form, allowing the anonymous use of their medical data.

### 2.2. Participants and Data Collection

In order to complete this retrospective study, the medical records of patients admitted to a tertiary hospital for oral and maxillofacial surgery in the north-west region of Romania were analyzed. The analyzed medical files belonged to patients who were admitted between 1 January 2002 and 31 December 2011 (a period of 10 years). All medical records were verified impartially by two authors to prevent a bias occurring (R.I.J. and A.E.M.), and all the collected data were centralized using Microsoft Excel software. It must be emphasized that not all the patients admitted in this hospital needed surgery, but the specific focus of the hospital is oral and maxillofacial surgeries.

Data were extracted from the medical records of patients hospitalized during the mentioned period, and the following variables were analyzed: type of facial fracture (maxillary, mandibular and combined); fracture amplitude (total and fissure); fracture displacement (with displacement and without displacement); associated lesions (hematoma, laceration and abrasion); type of treatment instituted (orthopedic, cerclage, osteosynthesis plates and combined); treatment evolution (favorable and unfavorable); complications after treatment (absent, osteitis and vicious consolidation). The ages of the patients (0–6 years, 7–12 years and 13–18 years) and their living environments (urban and rural) were also considered.

The following criteria were required for patients to be included in the study: patients aged 18 years or less; patients who had at least one facial fracture line at the time of admission; patients who had undergone imaging that confirmed the presence and trajectory of the fracture line; patients who benefited from fracture treatment in the host institution; patients who were followed-up for at least 8 weeks after intervention and treatment.

The following exclusion criteria were considered: patients aged 19 years or more; patients who did not have facial fractures; patients who did not undergo imaging to confirm the fracture line and its trajectory; patients who were initially treated in another hospital; patients who were followed-up for less than 8 weeks after treatment; patients who did not have complete information in the medical records. Patients for whom the consent for the anonymous use of medical data was not signed were also excluded.

### 2.3. Statistical Analysis

Microsoft Office Excel/Word 2013 (Microsoft, Redmond, WA, USA) and IBM SPSS Statistics 25 (IBM, Chicago, IL, USA) were used for the statistical analysis. Quantitative variables were reported as means with standard deviations or medians with interpercentile ranges, and Mann–Whitney U/Kruskal–Wallis H tests were used to compare the groups. Fisher’s Exact Tests were used to compare qualitative variables that were stated in absolute forms or as percentages. Data from the contingency tables were detailed using Z-tests with a Bonferroni correction.

## 3. Results

**Figure 1:**
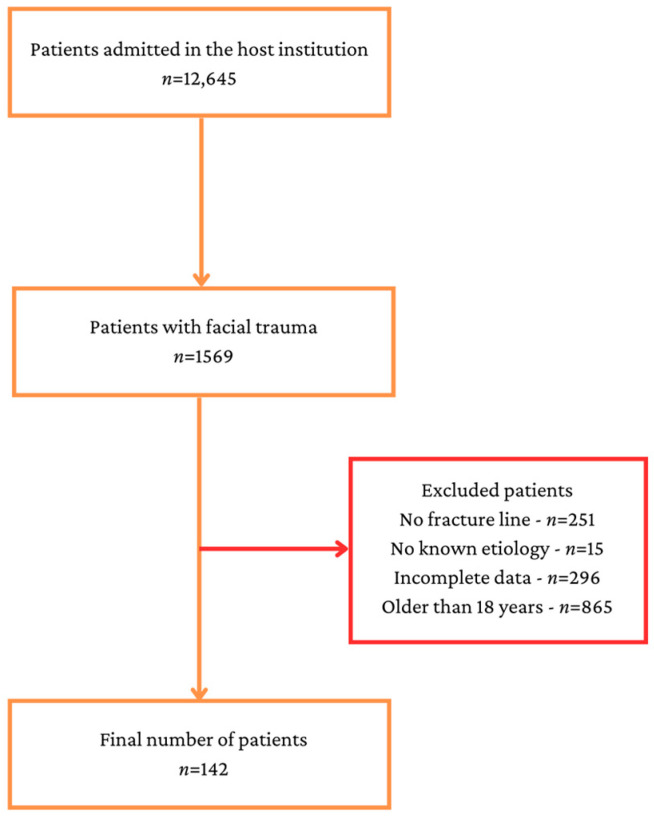
Study flowchart.

During the investigated period of time, 12,645 patients were admitted in the host institution, but after applying the inclusion and exclusion criteria, 142 patients remained in the study (Figure 1).

**Figure 2:**
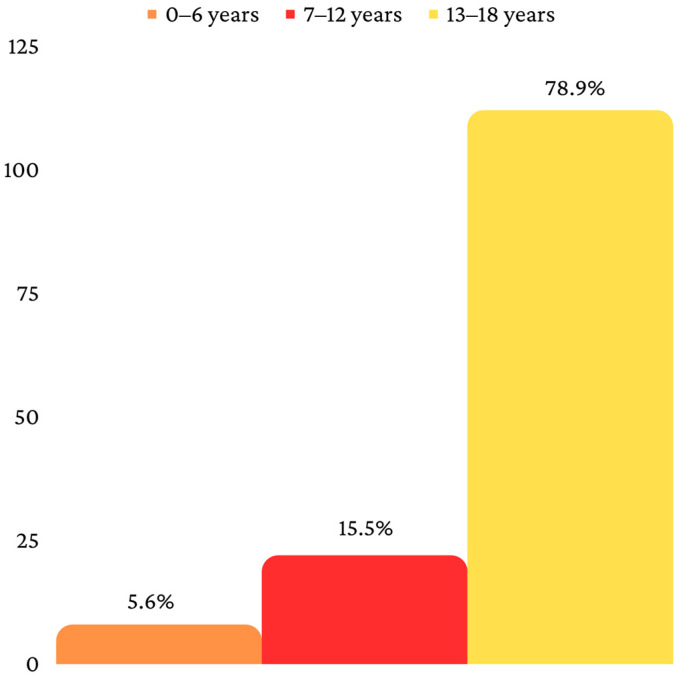
Distribution according to age.

**Figure 3:**
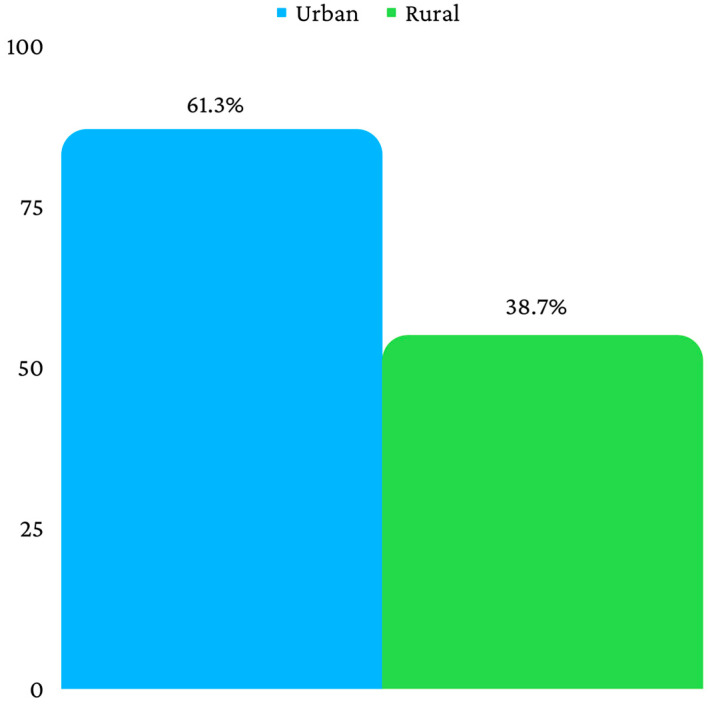
Distribution according to living environment.

**Figure 4:**
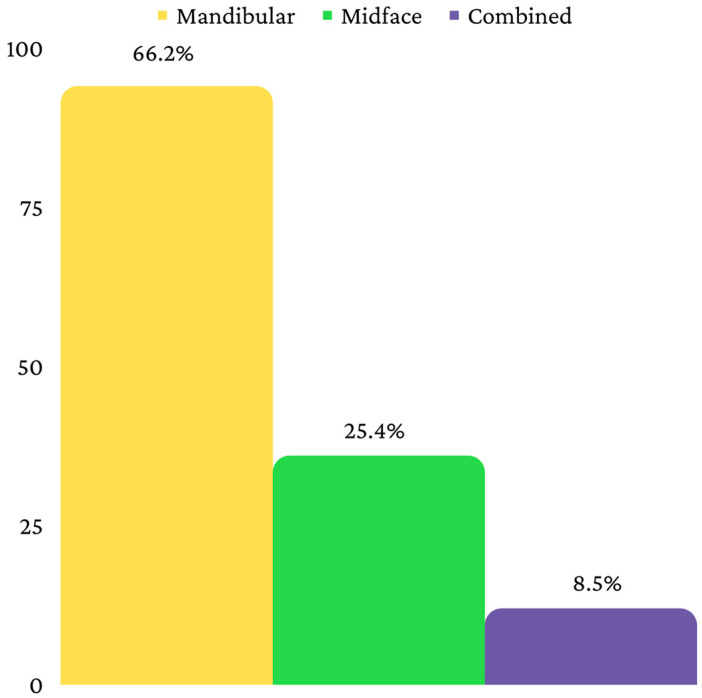
Distribution according to type of fracture.

The final sample consisted, therefore, of 142 patients, divided into three age categories, as follows: 0–6 years (*n* = 8), 7–12 years (*n* = 22) and 13–18 years (*n* = 112) (Figure 2). Fifty-five patients were from a rural environment, and eighty-seven were from an urban environment (Figure 3). Ninety-four patients had a mandibular fracture, thirty-six had a midface fracture and twelve had a combined fracture (Figure 4).

The mean age of the patients was 14.93 ± 3.75 years, with a median of 17 years.

Data in Table 1 show the distribution of patients according to the type of fracture and age/living environment. In the studied sample, mandibular fractures predominated in the 13–18 age group (84%), the proportion of which is similar to those of midface (69.4%) and combined (66.7%) fractures. However, in the 7–12 years age group, nine midface fractures (25% of all fractures) and four combined fractures (33.3% of the total number of combined fractures) were recorded. Among the patients from a rural environment, midface fractures were most frequently identified (52.8%), while among the patients from an urban environment, mandibular fractures accounted for more than half of the total number of patients living in an urban environment (62.8%). Patients living in the urban environment were more frequently associated with combined fractures than midface fractures (91.7% vs. 47.2%), while patients living in the rural environment were more frequently associated with midface fractures than combined fractures (52.8% vs. 8.3%).

Data in Table 2 show the locations of mandibular fractures. Fractures of the mandibular angle (50%) were most frequently diagnosed, followed by lateral fractures (34%), subcondylar fractures (33%) and paramedian fractures (28.70%). Only a small number of patients had fractures of the vertical ramus, median fractures or coronoid fractures.

Data in Table 3 show the locations of the midface fractures. Disjunctions of the malar bone were the most frequent ones (47.2%), followed by alveolar fractures (30.6%) and nasal fractures (22.2%). Le Fort I, II and III fractures were identified in six patients, while orbital floor fractures were diagnosed in five patients.

Most of the patients had total mandibular fractures (*n* = 93, 98.9%), midface fractures (*n* = 30, 83.3%) and combined fractures (*n* = 11, 91.7%). Patients with fissures were more frequently associated with midface fractures than they were with mandibular fractures (16.7% vs. 1.1%), while patients with total fractures were more frequently associated with mandibular fractures than they were with midface fractures (98.9% vs. 83.3%). Most mandibular fractures (*n* = 87, 92.6%) and midface fractures (*n* = 29, 80.6%) were without displacement, but for combined fractures, the distribution between displaced and non-displaced fractures was equal. Patients with displaced fractures were more frequently associated with combined fractures than they were with mandibular fractures (50% vs. 7.4%) (Table 4).

Data in Table 5 show the distribution of patients according to the type of fracture and the presence of associated lesions. Most patients diagnosed with a mandibular fracture (76.6%) did not present any hematoma, but they presented lacerations (83%) or abrasions (77.7%). A total of 80.6% of the patients diagnosed with midface fractures had an associated hematoma, and 91.7% of the patients diagnosed with combined fractures had an associated hematoma. The observed associations were statistically significant, and Z tests with Bonferroni correction detailed the following: patients with hematomas were more frequently associated with combined fractures or midface fractures than they were with mandibular fractures (91.7%/80.6% vs. 23.4%); patients with lacerations were more frequently associated with combined fractures or midface fractures than they were with mandibular fractures (58.3%/47.2% vs. 17%); patients with abrasions were more frequently associated with combined fractures or midface fractures than they were with mandibular fractures (83.3%/44.4% vs. 22.3%).

The instituted treatment was, in general, orthopedic, for all three types of fractures (mandibular—86.2%; midface—91.7%; combined—66.7%). Most patients had favorable evolutions of mandibular (*n* = 91, 96.8%), midface (*n* = 36, 100%) and combined (*n* = 11, 91.7%) fractures. Most fractures did not present any complications at the follow-up. According to the Fisher’s Exact Test, the associations between treatment choice, evolution and complications were not statistically significant (Table 6).

## 4. Discussion

The clinical patterns of pediatric facial fractures are diverse, are influenced by a multitude of factors, can have different degress of severity and various locations and can cause complications [23,24]. It was initially assumed that age, gender and living environment could have an impact on the clinical patterns of pediatric facial fractures among the analyzed sample of children and adolescents, and these variables were investigated. The different locations of mandibular and midface fractures, as well as the amplitude and the displacement of fracture fragments, were considered to be important for drawing valid conclusions regarding the clinical characteristic of pediatric facial fractures. Knowing about the various treatment approaches, the short-term evolution and the possible complications helped to outline a clear picture regarding the morbidity of these fractures.

Age has a great influence on the clinical characteristics of pediatric facial fractures [23]. The patients included in this study were distributed in three different age groups, 0–6 years, 7–12 years and 13–18 years, respectively. This type of age distribution was preferred because it includes three important stages of development (preschool, school and adolescence) [23]. At the same time, the distribution of the three age groups respects the chronology of primary, mixed and permanent dentition. Primary teeth begin to erupt immediately after birth [25], with the stage of deciduous dentition ending at the age of six, when permanent teeth begin to erupt [26]. With the onset of the eruption of permanent teeth, the stage of mixed dentition begins, which ends when all permanent teeth have been exfoliated and the second permanent molars erupt, which usually occurs at the age of 12 [26]. After all primary teeth have been exfoliated, the growth of permanent dentition begins [26]. In the current study, the most frequently affected patients were included in the 13–18 years age group, which is a situation that was similar to studies from other populations [27,28]. Ferreira et al. (2016) reviewed a total of 2071 pediatric facial fractures among a sample of 1416 patients. They divided the patients into six different age group, as follows: 0–3 years, 4–6 years, 7–9 years, 10–12 years, 13–15 years and 16–18 years. Eight hundred and seventy-nine patients (62%) belonged to the last two age groups (13–15 years and 16–18 years) [27]. Hoppe et al. (2014) investigated a sample of 285 patients with pediatric facial fractures. They distributed the patients into four different age groups, as follows: 0–2 years, 3–8 years, 9–14 years and 15–18 years. Most fractures were identified in the last age group (15–18 years).

The patients were also distributed according to the living environment, urban or rural ones, because it has been demonstrated that the living environment and environmental circumstances influence different aspects of pediatric trauma [29]. Fractures have a higher incidence among patients living in urban environments, which is probably due to the higher population density and a greater exposure to different sports [30], but also due to the high degree of community violence in the urban environment [31]. In this study, a predilection for fractures occurring among patients living in the urban environment was observed, as well, with more than half of the patients included in this study sample living in an urban environment.

Regarding the location, fractures can involve the mandible, the midface or they can be combined. The literature usually identifies pediatric mandibular fractures as the most common pediatric facial fractures [32,33]. The mandible is a U-shaped bone that has 13 muscle attachments for muscles that perform numerous functions. The innervation of the mandible is provided by the lower alveolar nerve and its branches [34]. Iida and Matsuya (2002) identified, among a sample of 174 pediatric patients from Osaka, that 56% of facial fractures involved the mandible. The most frequent locations of mandibular fractures were condylar fractures, followed by fractures in the canine region and mandibular angle fractures. Eleven percent of patients were diagnosed with midface fractures [32]. Mukhopadhyay S. (2018) identified 131 mandibular fractures among a sample of 89 children. The most frequent locations were the condylar region, the angle of the mandible, the parasymphysis, the body of the mandible and the symphysis [33]. In this study, mandibular fractures were the most frequent ones, as well, but the preferred locations were slightly different compared to those in the previously cited studies. Thus, in this study, most mandibular fractures occurred at the level of the mandibular angle, followed by lateral mandibular fractures, and only then by subcondylar fractures. The predominance of mandibular fractures is most likely due to the prominent position of the mandible in the facial skeleton [35]. Pediatric mandibular fractures are, in general, without displacement or with minimal displacement due to the high elasticity of the cortical bone [36]. This is in agreement with this study, where most mandibular fractures were not displaced.

Hematomas are among the associated lesions that are most frequently identified after facial fractures. They are produced by the extravasation of blood from the marrow into tissue spaces [37]. In the present study, hematomas were most frequently associated with midface and combined fractures. Chapman et al. (2009) reported orbital hematomas as frequent complications in fractures involving the orbital roof [38], and Ferreira et al. (2015) identified an association between midface and combined fractures and associated lesions such as hematomas or abrasions [39]. The results are similar to this study, where associated injuries such as hematomas, lacerations and abrasions were identified especially in midface and combined fractures.

The treatment of pediatric facial fractures offers various possibilities, and researchers must take into account the age of the child. Among younger children, non-surgical treatments are preferred due to their higher healing and remodeling capacity, but also in order to avoid the impairment of future craniofacial growth [7,40]. Along with the increase in age and severity of the fracture, the need for surgical interventions in the management of pediatric facial fractures also increases [41]. Among pediatric patients, open reduction and internal fixation increase the risk of intramaxillary teeth injuries, developmental disorders and the need for future surgical reinterventions [5], but the development of bio-resorbable plates has been proven to be effective in reducing the risks of the surgical management of pediatric facial fractures [42]. In this study, most of the mandibular, midface and combined fractures were treated conservatively and orthopedically, with favorable evolution. The high osteogenic potential of pediatric patients is one of the factors responsible for the favorable evolution of these fractures [43].

In a previously published paper, the authors aimed to identify the main etiology among this sample of patients and offered information regarding the epidemiology (age, gender, living environment, fracture line and associated soft tissue lesions) [44]. However, due to the large amount of information, it was considered that addressing all issues related to pediatric facial fractures in a single manuscript would make the reading experience hard and redundant. Clinical patterns, treatment, evolution and complications were, therefore, not presented in the first article. Although the sample remains the same, this paper presented a large amount of information regarding the aforementioned issues, which helps in understanding the different fracture patterns, their treatment approach and evolution. Etiology was not discussed since it was presented in the previous paper. It is the authors’ opinion that the present study provides valuable information regarding the clinical patterns of pediatric facial fractures in the investigated region. This information can be used for establishing predictable therapeutic protocols, but also for rapidly and correctly diagnosing pediatric facial fractures. The implementation of pediatric facial fracture prevention programs can also take into account the information presented in this study.

However, the study also has some limitations. The retrospective nature of this research may determine that the data recorded at the time of admission, as well as the data recorded at the follow-up, may be incorrect or incomplete. The unicentric approach used limits the number of cases to a single oral and maxillofacial surgery center, which makes it possible for cases registered and treated in other institutions to present differences among the investigated characteristics. Another limitation is the absence of a long-term follow-up, since in this study, only post-fracture healing was evaluated. The small number of children diagnosed with pediatric facial fractures can also be considered as a limitation.

## 5. Conclusions

Most pediatric facial fractures were recorded in the 13–18 years age group. Mandibular and combined fractures predominated among patients living in an urban environment, and midface fractures predominated among patients living in a rural environment. Most fractures were total fractures, but without the displacement of fractured fragments. Most patients with associated lesions such as hematomas, lacerations and abrasions were more frequently associated with midface or combined fractures than they were with mandibular fractures. For the majority of patients, the instituted treatment had a favorable evolution, without complications at the follow-up.

## Figures and Tables

**Table 1:** Type of fracture and age/age group/living environment.

Fracture (*n*, %)	Mandibular	Midface	Combined	
**Age**				*p* *
Mean ± SD	15.32 ± 3.63	14.02 ± 4.01	14.58 ± 3.75	0.139
Median (IQR)	17 (14–18)	16 (10.25–17)	16 (10.25)–18	
**Age Group**				*p* **
0–6 years	6 (6.4%)	2 (5.6%)	0 (0%)	0.067
7–12 years	9 (9.6%)	9 (25%)	4 (33.3%)	
13–18 years	79 (84%)	25 (69.4%)	8 (66.7%)	
**Living environment**				*p* **
Rural	35 (37.2%)	19 (52.8%)	1 (8.3%)	0.017
Urban	59 (62.8%)	17 (47.2%)	11 (91.7%)	

*n*—number; %—percentage; * Kruskal–Wallis H Test; ** Fisher’s Exact Test.

**Table 2:** Location of the mandibular fractures.

Fracture Location	No.	Percentage *
Mandibular angle	47	50%
Lateral	32	34%
Subcondylar	31	33%
Paramedian	27	28.70%
Vertical ramus	3	3.20%
Median	2	2.10%
Coronoid	2	2.10%

* out of the total number of mandibular fractures.

**Table 3:** Location of the midface fractures.

Fracture Location	No.	Percentage *
Disjunction of the malar bone	17	47.2%
Alveolar	11	30.6%
Nasal	8	22.2%
Orbital floor	5	13.9%
Le Fort I	2	5.6%
Le Fort II	2	5.6%
Le Fort III	2	5.6%
Malar dysfunction	17	47.2%

* out of the total number of mandibular fractures.

**Table 4:** Type of fracture and amplitude/displacement.

Fracture (*n*, %)	Mandibular	Midface	Combined	*p* *
**Amplitude**				
Fissure	1 (1.1%)	6 (16.7%)	1 (8.3%)	0.002
Total	93 (98.9%)	30 (83.3%)	11 (91.7%)	
**Displacement**				
No	87 (92.6%)	29 (80.6%)	6 (50%)	0.001
Yes	7 (7.4%)	7 (19.4%)	6 (50%)	

*n*—number; %—percentage; * Fisher’s Exact Test.

**Table 5:** Type of fracture and associated lesions.

Fracture (*n*, %)	Mandibular	Midface	Combined	*p* *
**Hematoma**				
No	72 (76.6%)	7 (19.4%)	1 (8.3%)	<0.001
Yes	22 (23.4%)	29 (80.6%)	11 (91.7%)	
**Laceration**				
No	78 (83%)	19 (52.8%)	5 (41.7%)	<0.001
Yes	16 (17%)	17 (47.2%)	7 (58.3%)	
**Abrasion**				
No	73 (77.7%)	20 (55.6%)	2 (16.7%)	<0.001
Yes	21 (22.3%)	16 (44.4%)	10 (83.3%)	

*n*—number; %—percentage; * Fisher’s Exact Test.

**Table 6:** Type of fracture and treatment/evolution/complications.

Fracture (*n*, %)	Mandibular	Midface	Combined	*p* *
**Treatment**				
Orthopedic	81 (86.2%)	33 (91.7%)	8 (66.7%)	0.085
Osteosynthesis plates	1 (1.1%)	1 (2.8%)	0 (0%)	
Cerclage	5 (5.3%)	0 (0%)	0 (0%)	
Combined	7 (7.4%)	2 (5.6%)	4 (33.3%)	
**Evolution**				
Favorable	91 (96.8%)	36 (100%)	11 (91.7%)	0.229
Unfavorable	3 (3.2%)	0 (0%)	1 (8.3%)	
**Complications**				
No complications	91 (96.8%)	36 (100%)	11 (91.7%)	0.124
Osteitis	3 (3.2%)	0 (0%)	0 (0%)	
Vicious consolidation	0 (0%)	0 (0%)	1 (8.3%)	

*n*—number; %—percentage; * Fisher’s Exact Test.

## Data Availability

The data presented in this study are available on request from the corresponding authors. The data are not publicly available due to privacy reasons.

## References

[1] Gómez Roselló E., Quiles Granado A.M., Artajona Garcia M., Juanpere Martí S., Laguillo Sala G., Beltrán Mármol B., Pedraza Gutiérrez S. (2020). Facial fractures: Classification and highlights for a useful report. Insights Imaging.

[2] Lalloo R., Lucchesi L.R., Bisignano C., Castle C.D., Dingels Z.V., Fox J.T., Hamilton E.B., Liu Z., Roberts N.L.S., Sylte D.O. (2020). Epidemiology of facial fractures: Incidence, prevalence and years lived with disability estimates from the Global Burden of Disease 2017 study. Inj. Prev..

[3] Juncar M., Tent P.A., Juncar R.I., Harangus A., Mircea R. (2021). An epidemiological analysis of maxillofacial fractures: A 10-year cross-sectional cohort retrospective study of 1007 patients. BMC Oral Health.

[4] Cole P., Kaufman Y., Hollier L.H. (2009). Managing the pediatric facial fracture. Craniomaxillofac. Trauma Reconstr..

[5] Braun T.L., Xue A.S., Maricevich R.S. (2017). Differences in the Management of Pediatric Facial Trauma. Semin. Plast. Surg..

[6] Imahara S.D., Hopper R.A., Wang J., Rivara F.P., Klein M.B. (2008). Patterns and outcomes of pediatric facial fractures in the United States: A survey of the National Trauma Data Bank. J. Am. Coll. Surg..

[7] Vyas R.M., Dickinson B.P., Wasson K.L., Roostaeian J., Bradley J.P. (2008). Pediatric facial fractures: Current national incidence, distribution, and health care resource use. J. Craniofac. Surg..

[8] Dobitsch A.A., Oleck N.C., Liu F.C., Halsey J.N., Hoppe I.C., Lee E.S., Granick M.S. (2019). Sports-Related Pediatric Facial Trauma: Analysis of Facial Fracture Pattern and Concomitant Injuries. Surg. J..

[9] Montovani J.C., de Campos L.M., Gomes M.A., de Moraes V.R., Ferreira F.D., Nogueira E.A. (2006). Etiology and incidence facial fractures in children and adults. Braz. J. Otorhinolaryngol..

[10] Gassner R., Tuli T., Hächl O., Moreira R., Ulmer H. (2004). Craniomaxillofacial trauma in children: A review of 3385 cases with 6060 injuries in 10 years. J. Oral Maxillofac. Surg..

[11] Andrew T.W., Morbia R., Lorenz H.P. (2019). Pediatric Facial Trauma. Clin. Plast. Surg..

[12] Grunwaldt L., Smith D.M., Zuckerbraun N.S., Naran S., Rottgers S.A., Bykowski M., Kinsella C., Cray J., Vecchione L., Saladino R.A. (2011). Pediatric facial fractures: Demographics, injury patterns, and associated injuries in 772 consecutive patients. Plast. Reconstr. Surg..

[13] Totonchi A., Sweeney W.M., Gosain A.K. (2012). Distinguishing anatomic features of pediatric facial trauma. J. Craniofac. Surg..

[14] Adibelli Z.H., Songu M., Adibelli H. (2011). Paranasal sinus development in children: A magnetic resonance imaging analysis. Am. J. Rhinol. Allergy..

[15] Shah R.K., Dhingra J.K., Carter B.L., Rebeiz E.E. (2003). Paranasal sinus development: A radiographic study. Laryngoscope.

[16] Alcalá-Galiano A., Arribas-García I.J., Martín-Pérez M.A., Romance A., Montalvo-Moreno J.J., Juncos J.M. (2008). Pediatric facial fractures: Children are not just small adults. Radiographics.

[17] Ryu J., Yun S.J., Lee S.H., Choi Y.H. (2020). Screening of pediatric facial fractures by brain computed tomography: Diagnostic performance comparison with facial computed tomography. Pediatr. Emerg. Care.

[18] Goldwasser T., Bressan S., Oakley E., Arpone M., Babl F.E. (2015). Use of sedation in children receiving computed tomography after head injuries. Eur. J. Emerg. Med..

[19] Nguyen B.N., Edwards M.J., Srivatsa S., Wakeman D., Claderon T., Lamoshi A., Wallenstein K., Fabiano T., Cantor B., Bass K. (2022). Clinical and radiographic predictors of the need for facial CT in pediatric blunt trauma: A multi-institutional study. Trauma Surg. Acute Care Open.

[20] Siy R.W., Brown R.H., Koshy J.C., Stal S., Hollier L.H. (2011). General management considerations in pediatric facial fractures. J. Craniofac. Surg..

[21] Ferreira P., Marques M., Pinho C., Rodrigues J., Reis J., Amarante J. (2004). Midfacial fractures in children and adolescents: A review of 492 cases. Br. J. Oral Maxillofac. Surg..

[22] Zimmermann C.E., Troulis M.J., Kaban L.B. (2006). Pediatric facial fractures: Recent advances in prevention, diagnosis and management. Int. J. Oral Maxillofac. Surg..

[23] Segura-Palleres I., Sobrero F., Roccia F., de Oliveira Gorla L.F., Pereira-Filho V.A., Gallafassi D., Faverani L.P., Romeo I., Bojino A., Copelli C. (2022). Characteristics and age-related injury patterns of maxillofacial fractures in children and adolescents: A multicentric and prospective study. Dent. Traumatol..

[24] Chao M.T., Losee J.E. (2009). Complications in pediatric facial fractures. Craniomaxillofac. Trauma Reconstr..

[25] Burgueño Torres L., Mourelle Martínez M.R., de Nova García J.M. (2015). A study on the chronology and sequence of eruption of primary teeth in Spanish children. Eur. J. Paediatr. Dent..

[26] Lynch R.J. (2013). The primary and mixed dentition, post-eruptive enamel maturation and dental caries: A review. Int. Dent. J..

[27] Ferreira P.C., Barbosa J., Braga J.M., Rodrigues A., Silva Á.C., Amarante J.M. (2016). Pediatric Facial Fractures: A Review of 2071 Fractures. Ann. Plast. Surg..

[28] Hoppe I.C., Kordahi A.M., Paik A.M., Lee E.S., Granick M.S. (2014). Age and sex-related differences in 431 pediatric facial fractures at a level 1 trauma center. J. Craniomaxillofac. Surg..

[29] Marek A.P., Nygaard R.M., Cohen E.M., Polites S.F., Sirany A.E., Wildenberg S.E., Elsbernd T.A., Murphy S., Dean Potter D., Zielinski M.D. (2018). Rural versus urban pediatric non-accidental trauma: Different patients, similar outcomes. BMC Res. Notes.

[30] Hedström E.M., Waernbaum I. (2014). Incidence of fractures among children and adolescents in rural and urban communities—Analysis based on 9965 fracture events. Inj. Epidemiol..

[31] Pittman S.K., Farrell A.D. (2022). Patterns of community violence exposure among urban adolescents and their associations with adjustment. Am. J. Community Psychol..

[32] Iida S., Matsuya T. (2002). Paediatric maxillofacial fractures: Their aetiological characters and fracture patterns. J. Craniomaxillofac. Surg..

[33] Mukhopadhyay S. (2018). A retrospective study of mandibular fractures in children. J. Korean Assoc. Oral Maxillofac. Surg..

[34] Panesar K., Susarla S.M. (2021). Mandibular Fractures: Diagnosis and Management. Semin. Plast. Surg..

[35] Gadicherla S., Sasikumar P., Gill S.S., Bhagania M., Kamath A.T., Pentapati K.C. (2016). Mandibular Fractures and Associated Factors at a Tertiary Care Hospital. Arch. Trauma Res..

[36] Pickrell B.B., Serebrakian A.T., Maricevich R.S. (2017). Mandible Fractures. Semin. Plast. Surg..

[37] Niazi K.T., Raja D.K., Prakash R., Balaji V.R., Manikandan D., Ulaganathan G., Yoganandha R. (2016). Massive expanding hematoma of the chin following blunt trauma. J. Pharm. Bioallied Sci..

[38] Chapman V.M., Fenton L.Z., Gao D., Strain J.D. (2009). Facial fractures in children: Unique patterns of injury observed by computed tomography. J. Comput. Assist. Tomogr..

[39] Ferreira P.C., Barbosa J., Amarante J.M., Carvalho J., Rodrigues A.G., Silva Á.C. (2015). Associated injuries in pediatric patients with facial fractures in Portugal: Analysis of 1416 patients. J. Craniomaxillofac. Surg..

[40] Kim S.H., Lee S.H., Cho P.D. (2012). Analysis of 809 facial bone fractures in a pediatric and adolescent population. Arch. Plast. Surg..

[41] Mckenzie J., Nguyen E. (2021). Minimally Invasive Surgical Management of Complex Pediatric Facial Fractures. Craniomaxillofac. Trauma Reconstr. Open.

[42] Singh G., Mohammad S., Chak R.K., Lepcha N., Singh N., Malkunje L.R. (2012). Bio-resorbable plates as effective implant in paediatric mandibular fracture. J. Maxillofac. Oral Surg..

[43] Naik P. (2021). Remodelling in Children’s Fractures and Limits of Acceptability. Indian J. Orthop..

[44] Țenț P.A., Juncar R.I., Moca A.E., Moca R.T., Juncar M. (2022). The Etiology and Epidemiology of Pediatric Facial Fractures in North-Western Romania: A 10-Year Retrospective Study. Children.

